# Defective Proventriculus Regulates Cell Specification in the Gastric Region of *Drosophila* Intestine

**DOI:** 10.3389/fphys.2020.00711

**Published:** 2020-07-14

**Authors:** Sonam Mehrotra, Priyanka Bansal, Neha Oli, Saraswathi Jayarajan Pillai, Sanjeev Galande

**Affiliations:** ^1^Advanced Centre for Treatment, Research and Education in Cancer, Kharghar, India; ^2^Department of Biology, Centre of Excellence in Epigenetics, Indian Institute of Science and Education and Research, Pune, India

**Keywords:** *Drosophila*, intestinal stem cells, cell specification, differentiation, Wnt signaling, copper cells

## Abstract

The gastrointestinal tract in metazoans consists of diverse epithelial cells with distinct cell morphology and physiological functions. The development and homeostasis of gastrointestinal epithelia involve spatiotemporal regulation by many signaling pathways, essential to confer their region-specific function and identity. The adult *Drosophila* midgut and the mammalian intestine share a high degree of conservation between such signaling pathways. Due to availability of sophisticated techniques for genetic manipulation, *Drosophila* is an excellent model to study mechanisms of tissue homeostasis regulation in a regionally defined manner. The gastric region located in the *Drosophila* middle-midgut coincides with the region containing fewest number of stem cells. It is also known as the copper cell (CC) region since it is composed of specialized groups of acid-secreting CCs, along with interstitial cells and enteroendocrine cells. The generation and maintenance of these cell populations are determined by the bone morphogenic protein-like Decapentaplegic (Dpp) signaling pathway. The morphogenic gradient of the Dpp signaling activity induces differential expression of specific transcription factors *labial* (*lab*) and *defective proventriculus* (*dve)*, which are required for the generation of various cell types specific to this region. In this study, we investigated the role of Dve in regulation of tissue homeostasis in the CC region. Our studies reveal that ectopic expression of *dve* in stem cells suppresses their self-renewal throughout the intestine. We further demonstrate that Dve is not required for generation of CCs. Higher levels of Dve can alter cell specification by inhibition of *cut* expression, which in turn prevents CC formation during homeostasis.

## Introduction

The small intestine consists of approximately 75% of the length of the gastrointestinal (GI) tract in humans; however, it is associated with a much lower rate of cancer occurrence as compared to the large intestine (Neugut et al., 1998). The potential factors that determine the susceptibility of a region in an organ to tumor formation are not completely understood. Studies utilizing murine models suggest that variations in the tissue microenvironment of different regions of the intestine contribute to this phenomenon (Haigis et al., 2004). Understanding mechanisms that regulate tissue homeostasis in a regionally defined manner will provide valuable insights into developing more effective strategies for other more common GI malignancies.

The epithelial lining the GI tract in both mammals and *Drosophila* originate from the endoderm. They exhibit intriguing similarities in terms of tissue morphology and physiological function. Recent findings suggest that there is high degree of conservation between the signaling pathways that regulate development, regeneration, and tissue homeostasis of the GI tract between mammalian and *Drosophila* (Apidianakis and Rahme, 2011). With the availability of sophisticated techniques for genetic manipulation and cell lineage analysis, the *Drosophila melanogaster* midgut serves as an excellent model to study adult intestinal stem cells (ISCs) during normal and pathological conditions.

**FIGURE 1:**
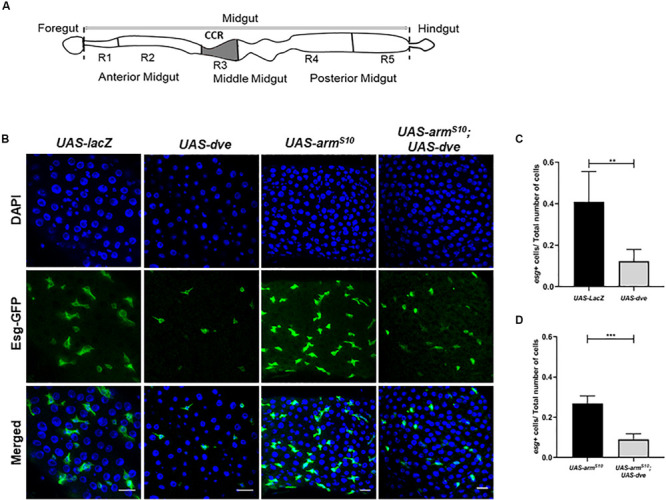
Ectopic expression of *dve* reduces the number of Esg^+^ stem and progenitor cells in adult *Drosophila* midgut. **(A)** Schematic diagram depicting different regions of *Drosophila* intestine from the anterior to the posterior end. The schematic of the midgut depicting the copper cell region and the region specifically regulated by the Dpp signaling pathway. **(B)** Representative immunofluorescence images of midguts (DAPI—blue and Esg-GFP—green) from the flies of the following genotypes: *esg^*ts*^* > *UAS-lacZ* as a control, *esg^*ts*^* > *UAS-dve*, *esg^*ts*^* > *UAS-arm^*S*10^*, and *esg^*ts*^* > *UAS-arm^*S*10^;UAS-dve*. Scale bar: 20 μm. **(C)** Quantification of esg^+^ cells (ISCs + EBs) in midguts from *esg^*ts*^* > *UAS-lacZ* as a control, *esg^*ts*^* > *UAS-dve*, flies. **(D)** Quantification of Esg^+^ cells (ISCs + EBs) in midguts from *esg^*ts*^* > *UAS-arm^*S*10^* and *esg^*ts*^* > *UAS-arm^*S*10^;UAS-dve* flies. Data presented as mean ± SEM calculated from *N* = 10 guts. **P ≤ 0.01 and ***P ≤ 0.001, calculated by Student’s two tailed *t* test.

The epithelial cells in different regions of the midgut share certain features but also possess distinct and highly specialized functions (Buchon et al., 2013; Marianes and Spradling, 2013). Based on the differences observed in terms of physiology and cell morphology along the anterior posterior axis, the *Drosophila* midgut can be divided into different regions, namely, anterior midgut (AM), middle midgut (MM), and posterior midgut (PM; Figure 1A; Marianes and Spradling, 2013). These regions can be further subdivided into specific compartments having unique histology and gene expression signatures. Detailed molecular characterization of these subregions has revealed variations in turnover rates of resident stem cells during homeostasis (Marianes and Spradling, 2013; Li and Jasper, 2016). The AM and PM exhibit higher number of resident stem cells; however, the MM coincides with the fewer number of resident stem cells that are mostly quiescent (Micchelli and Perrimon, 2006). During tissue homeostasis, regional boundaries and regional autonomy of resident stem cells are critically maintained (Marianes and Spradling, 2013). The daughter cells of a particular region strictly occupy the same compartment as the mother stem cell. Additionally, the stem cell-derived tumors do not cross regional boundaries (Driver and Ohlstein, 2014).

*Drosophila* midgut is maintained by an intricate balance between self-renewal and differentiation of multi-potent ISCs. These divide asymmetrically to renew and generate a transient pluripotent progenitor cell, enteroblast (EB), which differentiates into either nutrient absorptive enterocytes (EC) or secretory enteroendocrine (ee) cells (Micchelli and Perrimon, 2006; Ohlstein and Spradling, 2006; O’Brien et al., 2011). Tissue intrinsic factors such as Notch and insulin signaling pathways and exogenous factors such as pathogens, injury, and food uptake play critical roles in the decision between self-renewal and differentiation (Ohlstein and Spradling, 2007; Foronda et al., 2014). Moreover, the transcription factor Escargot (Esg), which is expressed in all ISCs, regulates the stem cell pool through the modulation of Notch activity (Beehler-Evans and Micchelli, 2015). Further, recent studies suggest that EC and ee are not generated from a common progenitor EB, but rather from a pre-committed ISC (Ohlstein and Spradling, 2007; Biteau and Jasper, 2014; Korzelius et al., 2014; Beehler-Evans and Micchelli, 2015; Guo and Ohlstein, 2015).

A stomach-like gastric region is located in the *Drosophila* MM. This consists of a specialized group of acid-secreting copper cells (CC) similar to the parietal cells of the mammalian stomach, along with interstitial cells and ee cells (Shanbhag and Tripathi, 2009; Strand and Micchelli, 2011). Due to the presence of copper cells, this region of the midgut is also known as the copper cell region (CCR; Figure 1A). Homeostasis in the gastric region is maintained by a population of gastric stem cells (GSSC) (Wang et al., 2014). These are generally quiescent but respond to environmental challenges and can be induced to divide asymmetrically to self-renew and generate a transient pluripotent gastroblast (GB). The GB is capable of giving rise to all types of cells in the CCR (Strand and Micchelli, 2011). During embryogenesis, the generation of various cell populations in the gastric region is determined by the bone morphogenic protein (BMP)-like Decapentaplegic (Dpp) signaling pathway. The secretion of Dpp at the boundary between the PM and MM results in a morphogen gradient in the MM, which induces a high threshold target *labial* (*lab*) and a low threshold target *defective proventriculus* or (*dve*) (Nakagoshi, 2005). The regional boundaries that are established during embryogenesis are thus maintained by established patterns of specific transcription factors, which in turn determine the specification of cells generated from the progenitor cell GB (Dutta et al., 2015a; Dutta et al., 2015b). The GBs further differentiate into either of the three different cell types: Labial^+^/Cut^+^ Copper cells, Dve^+^/Cut^–^ interstitial cells, and Pros^+^ ee cells, respectively. Recent studies have highlighted mechanisms that regulate the Dpp signaling pathway, GSSC proliferation, and differentiation in the CCR (Li et al., 2016). A study demonstrated that ectopic expression of a Dpp target *labial* in all Esg^+^ cells including both ISCs and GSSCs was sufficient to block their proliferation. On the other hand, downregulation of *labial* expression did not change turnover rates of either GSSCs and ISCs (Dutta et al., 2015a; Dutta et al., 2015b). Hence, Labial is required for generation of copper cells in the CCR (Dubreuil, 2004; Li et al., 2016). The functions of the other Dpp target Dve in the CCR remain poorly understood.

In this study, we investigated the specific role played by Dve during tissue homeostasis within and beyond the CCR boundary. Further, we characterized the function of Dve toward cell specification in adult *Drosophila* MM. Our studies reveal that ectopic expression of *dve* in Esg^+^ stem cells suppress their self-renewal throughout the midgut. In the gastric region/CCR, Dve suppresses the proliferation of induced GSSCs. Depletion of Dve did not prevent generation of Cut^+^ copper cells from induced proliferation of GSSC and overexpression of Dve reduced the number of Cut positive cells. These data suggest that Dve is not required for copper cell generation, and high levels of Dve can alter cell specification by preventing copper cell formation.

## Results

### Dve Expression Is Sufficient to Reduce Esg^+^ Stem Cell and Progenitor Cell Populations

To study the role of Dve in stem cell maintenance and tissue homeostasis in the CCR, the binary Gal4/UAS system was employed. We ectopically expressed *UAS-dve* using a conditional temperature-sensitive *esg-Gal4, UAS-GFP;tub-Gal80^*ts*^* (henceforth referred to as *esg-Gal4^*ts*^*). This allowed the expression of *dve* in stem cells (ISCs and GSSCs) and their non-proliferative progeny of progenitor cells (EBs and GBs) in the midgut, all of which were identified by GFP expression. The number of Esg^+^ cells were then estimated in the midguts of *UAS-dve/esg-Gal4^*ts*^* and *UAS-lacZ;esg-Gal4^*ts*^* (control flies). The number of Esg^+^ cells was significantly reduced in *UAS-dve*/*esg-Gal4^*ts*^* flies as compared to the control flies, suggesting that ectopic expression of *dve* decreased the population of Esg^+^ stem cells and progenitor cells (Figures 1B,C and Supplementary Figure S1). Further, the number of Esg^+^ cells was estimated upon ectopic expression of *dve* in genetic backgrounds, which are known to increase the proliferation of stem cells. These include constitutively activated Wnt or EGF pathways (Lin et al., 2008; Lee et al., 2009; Strand and Micchelli, 2011; Xu et al., 2011). For constitutive activation of the Wnt pathway, a mutant version of activated β-catenin homolog called *armadillo* referred to as *arm^*S*10^* was ectopically expressed in the midgut by using *UAS-arm^*S*10^*; *esg-Gal4^*ts*^* flies (Tolwinski and Wieschaus, 2004). Activation of the Wnt pathway increased the total number of cells in the midgut; however, the ratio of different cell populations did not alter significantly (Figure 1D). For example, the ratio between Esg^+^ cells to the total number (DAPI^+^) of cells in the midgut remained the same (Figures 1C,D). Ectopic expression of *dve* in flies with the activated Wnt pathway in *UAS-arm^*S*10^;UAS-dve*/*esg-Gal4^*ts*^* flies yielded a reduction in the number of Esg^+^ cells (Figure 1D). Activation of the EGF pathway by overexpression of mutant version of the *Ras* gene in *UAS-Ras 85D; esg-Gal4^*ts*^* flies exhibited a similar phenotype. This caused an increase in the total number of (DAPI^+^) cells in the midgut (Supplementary Figure S2); however, the ratio of Esg^+^ cells to the total number of cells (DAPI^+^ cells) did not change significantly. The number of Esg^+^ cells was similar to that observed in *UAS-lacZ*; *esgGal4*^*ts*^ control flies. Expression of *dve* was sufficient to decrease the ratio of Esg^+^ cell population in the presence of the activated EGF pathway in *UAS-Ras85D;UAS-dve*/*esg-Gal4^*ts*^* flies (Supplementary Figure S2). Together, these results suggest that the ectopic expression of *dve* is sufficient to reduce the number of resident stem cells and progenitors throughout the midgut beyond the boundary of the gastric region.

### Ectopically Induced Dve Does Not Induce Cell Death in ISCs/EBs

**FIGURE 2:**
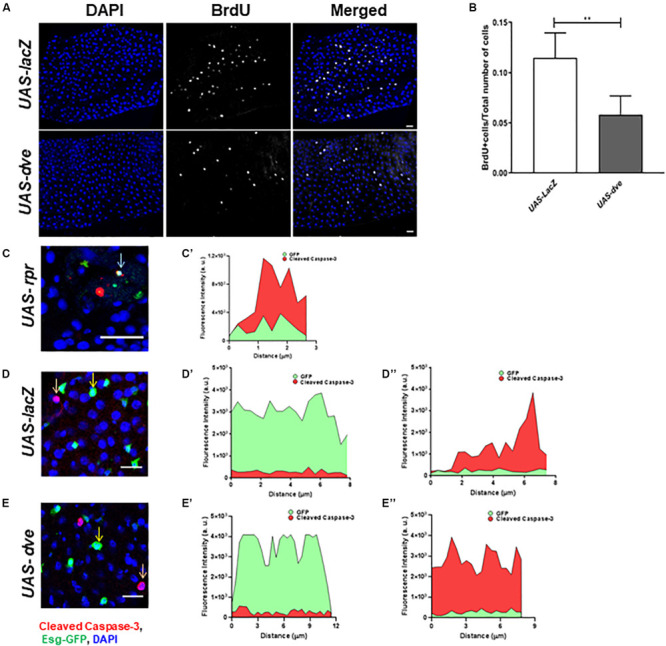
Overexpression of *dve* prolongs cell cycle but does not induce cell death. **(A)** Representative immunofluorescence images (DAPI—blue and BrdU—gray) of midguts from *esg^*ts*^* > *UAS-lacZ* (control) and *esg^*ts*^* > *UAS-dve* flies. Scale bar: 20 μm. **(B)** Quantification of BrdU^+^ cells in *esg^*ts*^* > *UAS-lacZ* and *esg^*ts*^* > *UAS-dve* flies. Data presented as mean ± SEM calculated from *N* = 10 guts. **P ≤ 0.01 calculated by Student’s two-tailed *t* test. **(C,D)** Representative immunofluorescence images of midgut labeled with DAPI—blue, Esg-GFP—green, and apoptotic marker cleaved caspase 3—red. **(C,C′)** The graph depicts fluorescent intensities of red (cleaved caspase-3) and green (Esg-GFP) signals in cells marked by arrow in panel **(C)**. The overlapping red and green signals suggest expression of cleaved caspase-3 in cells overexpressing *rpr* in midguts from *esg^*ts*^* > *UAS-LacZ* (Positive control) flies. Representative image from midgut from panel **(D)** *esg^*ts*^* > *UAS-lacZ* (Negative control) and **(E)** *esg^*ts*^* > *UAS-dve* flies. The graphs depict the fluorescent intensities of red and green signals in cells marked by **(D′,E′)** yellow and **(D″,E″)** orange arrows in respective images **(D,E)**. The red and green signals in each of these cells do not overlap, suggesting that Esg-GFP + cells indicated in these images do not express cleaved caspase-3. Scale bar: 20 μm.

To determine the effect of Dve expression on ISC turnover, we estimated the number of proliferating cells by labeling the midguts with a thymidine analog BrdU and compared BrdU^+^ cells in *UAS-dve*/*esg-Gal4^*ts*^* and *UAS-lacZ*;*esg-Gal4^*ts*^* (control) flies. Ectopic expression of *dve* decreased the number of BrdU^+^ cells as observed in *UAS-dve*/*esg-Gal4^*ts*^* flies (Figures 2A,B), suggesting that ectopically induced Dve either delays or inhibits cell cycle progression of ISCs.

Furthermore, to determine if the ectopic expression of *dve* in Esg^+^ cells induces their cell death, we examined cleaved caspase-3 levels in GFP^+^ cells upon ectopic expression of *dve* or proapoptotic gene *reaper* in *UAS-dve*;*esg-Gal4^*ts*^* and *UAS-rpr*;*esg-Gal4^*ts*^* (positive control) flies, respectively. We also examined apoptotic cells in the midgut from *UAS-lacZ*;*esg-Gal4^*ts*^* as a negative control. The red (cleaved caspase-3) and the green (GFP) fluorescent intensities were measured in midgut cells from the respective genotype flies (Figures 2C–E). In *UAS-rpr*;*esg-Gal4^*ts*^* (control) flies, fewer GFP^+^ cells were observed due to higher cell death. Most GFP^+^ cells observed in *UAS-rpr*;*esg-Gal4^*ts*^* flies also exhibited cleaved caspase-3 staining, suggesting induction of apoptosis due to ectopic expression of *rpr* (Figures 2C,C’). The expression of cleaved caspase-3 was virtually undetectable in the GFP^+^ cells in *UAS-lacZ*;*esg-Gal4^*ts*^* (D–D″) and *UAS-dve*;*esg-Gal4^*ts*^* (E–E″) flies. The red and green fluorescent signals were not observed in the same cell. This confirms that the EsgGFP^+^ cell did not express cleaved caspase-3 upon ectopic expression of Dve in ISCs and EBs (Figures 2E–E”). Taken together, these data show that ectopically expressed Dve does not induce apoptosis in the Esg^+^ cells.

### Ectopic Expression of Dve in Esg^+^ Cells Reduces the Expression of Stem Cell Markers and Progenitor Cell Markers

The ISC pool is regulated by the modulation of the Notch signaling pathway, which is regulated by the transcription factor Escargot (Beehler-Evans and Micchelli, 2015).

**FIGURE 3:**
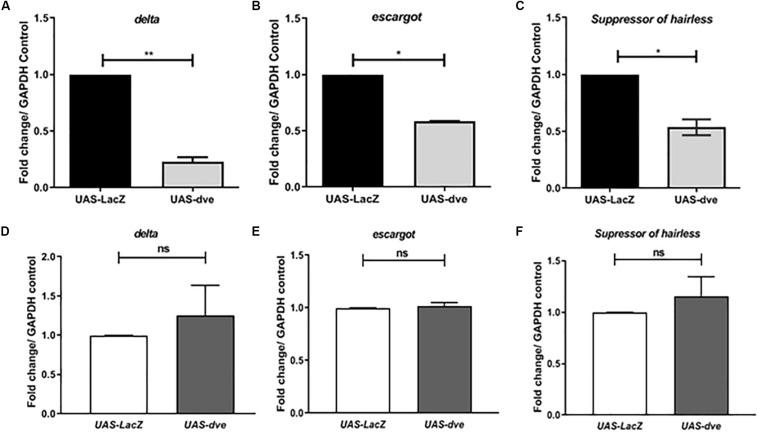
Ectopic expression of Dve in the midgut decreases the expression of stem cell and progenitor cell markers. **(A–F)** The transcript levels of *delta* (ISC marker), *escargot* (marker for both ISC and EB), and *Suppressor of Hairless* (EB marker) examined from midgut tissue from *esg^*ts*^* > *UAS-lacZ* (control) and *esg^*ts*^* > *UAS-dve* flies using quantitative RT-PCR. Data presented as fold change in mRNA levels. Relative mRNA expression of **(A)** *delta*, **(B)** *escargot*, and **(C)** *Suppressor of hairless* after 3 days of induction at 29°C. *N* = 3 (number of experiment sets); *P ≤ 0.05 and **P ≤ 0.01 calculated by Student’s two-tailed *t* test. **(D–F)** Relative mRNA expression of **(D)** *delta*, **(E)** *escargot*, and **(F)** *Suppressor of hairless* was estimated after 14 days aging post induction at 29°C. *N* = 3; ns (not significant) and *P* ≥ 0.05 calculated by Student’s two-tailed *t* test.

Suppression of *Escargot* and *Delta* expression in ISCs prevents the activation of the Notch pathway and in turn promotes their differentiation to non-proliferative cell types. The ISC cells divide asymmetrically to generate an ISC and EB, both of which are Esg+ cells. These cells are observed as a pair. In the presence of ectopic expression of Dve, the ISC and EB cells are not observed as pairs, but instead as single cells. The morphology of Esg^+^ cells seems to be altered, which may be due to the suppression of ISC/EB marker Escargot, which is required for maintenance of stem cell identity and suppressing differentiation. The exact mechanism by which ectopic expression of Dve leads to a change in the morphology of ISCs remains to be investigated (Korzelius et al., 2014; Wang et al., 2014). To examine the effect of *dve* expression in ISCs/EBs, we estimated the transcript levels of ISC and progenitor cell markers, *delta*, *esg*, and *Su(H)*, in *UAS-dve*/*esg-Gal4^*ts*^* flies 3 days after induction (Figures 3A–C) by using quantitative RT-PCR. These analyses revealed that the transcript levels of these specific factors decreased in midguts of *UAS-dve*/*esg-Gal4^*ts*^* as compared to those observed in *UAS-lacZ*/*esg-Gal4^*ts*^* flies. These results suggest that ectopically expressed Dve is sufficient to decrease the expression levels of *delta*, *escargot*, and *Su(H)*. The expression of these ISC/EB markers was, however, restored to normal levels after 14 days after induction of ectopic *dve* expression (Figures 3D–F). These results suggest that the presence of Dve in stem cell can alter the levels of stem cell markers, which indirectly can regulate its stem cell identity.

### High Levels of Dve Can Alter Products of ISC/EB Differentiation in the Presence of the Activated Wnt Pathway

**FIGURE 4:**
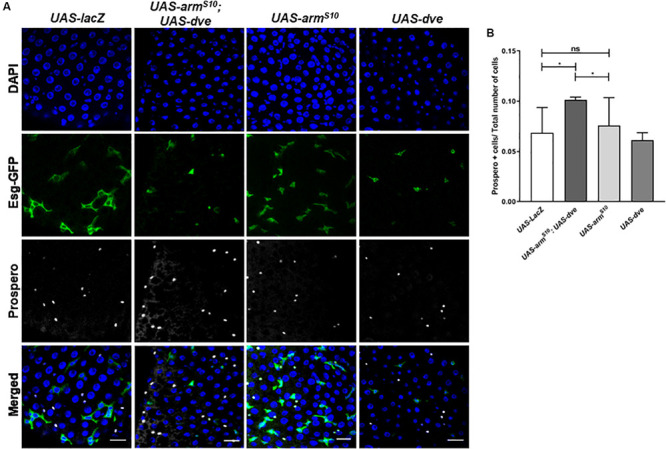
Ectopic expression of *dve* increases the number of differentiated enteroendocrine cells in the presence of the constitutively activated Wnt pathway. **(A,B)** Estimation of Prospero^+^ enteroendocrine (ee) cells by immunostaining with Prospero antibody. **(A)** Representative immunofluorescence images of midguts (DAPI—blue, Esg-GFP—green, and Prospero—gray) from flies of the following genotypes: *esg^*ts*^* > *UAS-lacZ* as a control, *esg^*ts*^* > *UAS-dve, esg^*ts*^* > *UAS-arm^*S*10^* and *esg^*ts*^* > *UAS-arm^*S*10^;UAS-dve*. Scale bar: 20 μm. **(B)** Quantification of Prospero^+^ cells in midguts from *esg^*ts*^* > *UAS-lacZ* as a control, *esg^*ts*^* > *UAS-dve, esg^*ts*^* > *UAS-arm^*S*10^* and *esg^*ts*^* > *UAS-arm^*S*10^;UAS-dve* flies. *N* = 10 (number of midguts analyzed per sample) *P < 0.05 (Student’s two-tailed *t* test).

Previous studies have demonstrated the requirement of Wnt activation for cell specification and regulation of ISC proliferation during homeostasis (Tian et al., 2016; Yang et al., 2016). Upon constitutive activation of the Wnt pathway in the *UAS-arm^*S*10^; esg-Gal4^*ts*^* flies, we observed an overall increase in the total number of cells without causing a significant change in the proportions of ISCs and EBs. The 90–95% of the total cell population is composed of EC. These can be identified by their morphology inclusive of a large polyploid nucleus. Since EC constitute majority of the cells in the midgut, it was difficult to estimate subtle changes in their numbers due to any genetic manipulation. Therefore, to investigate the effect of Wnt activation on the number of differentiated cell population, we quantified the number of prospero^+^ ee cells in both *UAS-arm^*S*10^;esg-Gal4^*ts*^*, and *UAS-arm^*S*10^;UAS-dve*/*esg-Gal4^*ts*^* flies, respectively (Figure 4A). The total number of cells (DAPI^+^ cells) increased in *UAS-arm^*S*10^;esg-Gal4^*ts*^* flies as compared to *UAS-lacZ; esg-Gal4^*ts*^* flies. The proportion of ee cells with respect to the total number of cells, however, did not change significantly (Figure 4B). It is possible that ectopic expression of *UAS-arm^*S*10^* gave rise to a higher number of EC in the midgut. The proportion of ee cells in the midgut from *UAS-arm^*S*10^; UAS-dve*/*esg-Gal4^*ts*^* increased as compared to those estimated in the midgut of *UAS-arm^*S*10^; esg-Gal4^*ts*^* flies (Figure 4B). In a similar experiment, the number of prospero^+^ cells was estimated upon activation of the EGF pathway in *UAS-Ras85D;esg-Gal4^*ts*^* and *UAS-Ras85D;UAS-dve*/*esg-Gal4^*ts*^* flies (Supplementary Figure S3A). The total number of (DAPI+) cells increased upon activation of the EGF pathway, but the proportion of differentiated ee cells did not change in *UAS-Ras85D;UAS-dve*/*esg-Gal4^*ts*^* flies as compared to *UAS-Ras85D;esg-Gal4^*ts*^* flies (Supplementary Figure S3B). These data suggest that constitutive activation of both Wnt or EGF signaling pathways increases the total number of cells, but the proportion of different cell populations is maintained. Ectopic expression of Dve only in the presence of the constitutively active Wnt signaling pathway increases the proportion of ee cells. How Dve alters cell specification of differentiated cells in the presence of the activated Wnt pathway is a question that remains to be investigated.

### High Levels of Dve Can Inhibit Generation of Cut^+^ Cells Upon Induced Proliferation of Stem Cells in the Gastric Region

**FIGURE 5:**
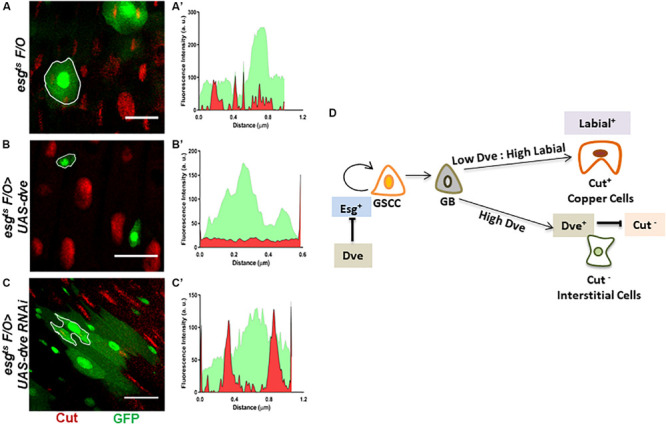
Perturbations of Dve levels can alter cell specification in the copper cell region. **(A–D)** Generation of mitotic clones with either depletion or overexpression of *dve* by using intestinal cell lineage tracing system. Representative immunofluorescence images of mitotic clones generated in copper cell region of midguts (Esg-GFP—green and Cut—red) from the flies of the following genotypes **(A)** *esg^*ts*^ F/O* as control, **(B)** *esg^*ts*^ F/O* > *UAS-dve*, and **(C)** *esg^*ts*^ F/O* > *UAS-dve^*RNAi*^*. The fluorescent intensities of red and green signals representing Cut and GFP expression in the mitotic clones indicated with white outline in the respective panels have been presented in graphs **(A′)** *esg^*ts*^ F/O* (control) clones, **(B′)** *esg^*ts*^ F/O* > *UAS-dve*, and **(C′)** *esg^*ts*^ F/O* > *UAS-dve^*RNAi*^*. Overlap between red and green signals indicate expression of both Cut and GFP in the outlined clone. Scale bar: 5 μm. **(D)** Schematic diagram depicting the role of Dpp targets Labial and Dve in cell specification in the copper cell/gastric region of the *Drosophila* midgut.

Dve is a low-threshold target of the Dpp signaling pathway that is expressed specifically in the CCR. The temporal regulation of Dpp morphogen gradient is critical as it is capable of transforming midgut cells to copper cells only during pupation but not in adults (Driver and Ohlstein, 2014). It has been reported that ectopic expression of *labial* in the AM-induced Cut^+^ copper cells and *labial* knockdown prevented the formation of copper cells in the CCR (Marianes and Spradling, 2013). Therefore, while the role of Labial in specification of copper cells has been established, the role of Dve in cell specification in CCR is not clearly understood. To investigate this, we used the intestinal lineage tracing system, which utilizes the temperature-inducible expression of a FLIP recombinase or FLPase and an Act > STOP > Gal4 cassette. The expression of the FLPase was induced using an *esg-Gal4^*ts*^* driver that activates a constitutive Act > STOP > Gal4 driver by removing the “STOP” sequence flanked by FRT sites on both sides. This system was used to generate clones (hereby referred to as esg^*ts*^F/O clones) that were identified by GFP expression. These clones expressed either UAS-*dve*^*RNAi*^ in induced GSSCs or their descendent progeny (Kolzer et al., 2003; Ohlstein and Spradling, 2007). We specifically analyzed clones in the CCR. The fluorescent intensities for GFP and Cut were measured in mitotic clones obtained for esg^*ts*^F/O (control), esg^*ts*^F/O > UAS-*dve*, and esg^*ts*^F/O > *UAS-dve^*RNAi*^* depicted as outlined GFP^+^ clones (Figures 5A–C). esg^*ts*^F/O clones showed overlapping signals of GFP and Cut, suggesting that these clones contained Cut^+^ cells (Figures 5A,A’). On the other hand, esg^*ts*^F/O > UAS-*dve* mitotic clones were very small, containing between one and two cells. These exhibited only GFP signal and lacked Cut signal as shown in the fluorescent intensity plot (Figures 5B,B’). This suggests that esg^*ts*^F/O clones overexpressing *dve* lacked any Cut^+^ cells. In comparison, mitotic clones expressing esg^*ts*^F/O > *UAS-dve^*RNAi*^* were larger and contained many more cells and exhibited expression of both GFP and Cut. This suggested that these clones contained Cut^+^ cells (Figures 5C,C’). Taken together, these results suggest that the overexpression of *dve* suppresses proliferation of induced GSSCs. The data further reveal that depletion of *dve* does not prevent the formation of Cut^+^ cells; however, the overexpression of *dve* directly or indirectly inhibits Cut expression. It is possible that high levels can alter cell specification during homeostasis in the gastric region indirectly by inhibition of Cut expression. The identity of Cut^+^ cells observed in the mitotic clones was not determined and requires further study in the future.

## Discussion

The intestinal epithelium undergoes high turnover. It is essential that homeostasis is critically regulated in such tissues to prevent hyperplasia and/or metaplasia. Additionally, the regeneration of cell lineages has to be precisely regulated in order to maintain the identity and function of respective regions in the intestine. Studies suggest that the number of resident stem cells, their renewal, and differentiation are regulated in a region-specific manner. Also, the stem cell niches of different regions are possibly adapted to the physiology of that compartment (Li and Jasper, 2016).

The cell lineages in the CCR are maintained by the differentiation of a small number of resident stems of this region, the GSSC. The differentiation of the GSSC is controlled by many signaling pathways including the Dpp signaling pathway (Marianes and Spradling, 2013). Perturbations of the Dpp signaling can result into metaplasia-like phenotypes where Cut^+^ copper cell undergoes trans-differentiation to generate Pdm1^+^ Enterocyte. The Dpp signaling is further regulated by Mad/Dad signaling, which fine-tunes the self-renewal and differentiation of GSSCs (Li et al., 2016). The Dpp gradient thus established induces expression of a high threshold target (Labial) and a low threshold target (Dve) (Strand and Micchelli, 2011). Previously, it has been shown that the ectopic expression of Dpp or Labial is sufficient to generate copper cells in the AM but not in the PM (Dubreuil, 2004; Li et al., 2016). In this study, we investigated the role of Dve, the low-threshold target of Dpp and a transcription factor specifically expressed in the CCR during tissue homeostasis within and beyond the CCR boundary. Our results demonstrated that ectopic expression of *dve* in esg^+^ ISCs was sufficient to block their proliferation and self-renewal throughout the midgut, including regions beyond the gastric region boundary. This suggests that Dve expression can inhibit proliferation of ISCs indirectly by suppression of Esg, which is required for maintenance of stem cells.

### Dve Is Not Required for Copper Cell Generation and Inhibits Cut Expression

As established earlier by Strand and Micchelli, the Esg^+^ GSSCs are normally quiescent but can be induced to generate all the cell lineages of the CCR. Here, we have induced Esg^+^ GSSC by heat stress and used an intestinal cell lineage tracing system to analyze the generation of cell types. We have shown that varying levels of Dve affect the number of different cell types during the regeneration process. This suggests that the levels of Dve are critical such that low levels or absence of Dve and high Labial levels induce copper cells. In contrast, high levels of Dve suppress Cut expression that directly or indirectly prevents the formation of Cut^+^ copper cells irrespective of the levels of Labial (Figure 5D). These results provide evidence in favor of the notion that only Labial expression is required and sufficient for Cut expression and generation of copper cells. Dve negatively regulates Cut expression, which in turn alters cell specification by inhibiting copper cell formation.

### Dve May Regulate Wnt Signaling Within the CCR Compartment

Multiple studies have indicated the requirement of Wnt/Wg signaling for maintenance of adult stem cell populations such as those found in the proventriculus, PM, hindgut, and CCR (Lin et al., 2008; Lee et al., 2009; Xu et al., 2011). Recent studies reveal that the activation of the Wnt signaling peaks near the compartment boundaries and low level is observed throughout the midgut compartments in both larval and adult stages. This gradient of Wg protein is known to be required for specification of cell fate and proliferation of resident stem cells within the compartment (Tian et al., 2016). During wing development in the third instar wing disks, Dve is required for restricting Wingless signaling at the Dorso-ventral (DV) boundary. The ectopic expression of Dve at the DV boundary in the wing disk is sufficient to disrupt the expression of Wg in the wing disk (Kolzer et al., 2003). It remains to be investigated if Dve plays a similar role in restricting the Wg protein to the CCR compartment boundaries. Wg protein has been shown to be dispensable for proliferation of GSSCs. The long-range effects of Wnt signaling and its crosstalk with the Dpp signaling pathway may play a role in the GSSC differentiation by mechanisms that are not completely understood (Tian et al., 2019). Further, how the crosstalk between the components of Dpp and other signaling pathways regulates the physiological identity of CCR remains to be investigated. Thus, this study provides new insights into the role of Dve in cell specification and stem cell renewal in a regionally defined manner in the *Drosophila* midgut. This knowledge will also be relevant in understanding mechanisms that determine susceptibility of different intestinal regions to tumor formation.

## Materials and Methods

### Fly Lines and Husbandry

Flies were maintained on standard media. Crosses were cultured at either 18 or 25°C in humidity-controlled incubators. Only female flies were analyzed in all experiments. The following fly strains were used: *UAS-Nuclear LacZ*, *UAS-dve* (BL 7086), *UAS-labial* (BL 7300), *Y^*I*^w^*III*8^, UAS-armS10* (BL 4782), *w, UAS-Ras85D* (BL 5788), *w; Kr/CyO; D/TM3-ser* (BL 7198), *UAS-reaper, UAS-LacZ* (G. Ratnaparkhi laboratory), *w; IF/CyO; MKRS/TM3-ser* (G. Ratnaparkhi laboratory), *w; esg-Gal 4,UAS-GFP, Tub-Gal 80^*ts*^/CyO* (*esg^*ts*^*) (N. Perrimon Laboratory), *w*; *esg-Gal4*, *tub-Gal80ts, UAS-GFP; UAS-FLP, Act* > *CD2* > *Gal4(UAS-GFP)/TM6B* (B. Edgar Laboratory), and *UAS-dveRNAi* (KK109538, VDRC). All stocks were obtained from the Bloomington stock center unless otherwise indicated.

### Temperature Shift Experiments

Crosses were established and cultured at 18°C, the permissive temperature, until adulthood. The collected progeny was aged for 5–7 days at 18°C and shifted to 29°C for 3 days for induction. Then, adult flies were kept on 5% glucose at 18°C for 5 h, prior to dissections. For aging experiments, after induction, the flies were aged for total 14 days post induction. For the *reaper*-induced cell death experiment, flies overexpressing *reaper* were collected and aged for 5–7 days at 18°C and then shifted to 29°C for 5–6 h before examination. Cultures were transferred onto fresh food augmented with yeast paste every 2–3 days during the experimental period.

### Quantification of ISCs + EBs in the Midgut Region

The binary Gal4/UAS system was employed to ectopically express candidate genes of interest. The candidate genes were expressed using *UAS* lines for each and a conditional temperature-sensitive *esg-Gal4 UAS-GFP;tub-Gal80^*ts*^* (henceforth referred to as *esg-Gal4^*ts*^*) Gal4 driver line. This allowed the expression of the specific gene in the ISCs and their non-proliferative progeny EBs, both of which were identified by GFP expression (represent *esg*+ cells) in the adult intestine. For estimation of GFP^+^ cells in each gut, 10–15 images spanning major regions of midgut (AM, MM, and PM, respectively) were captured and GFP^+^ cells were estimated in each image and added to calculate the total GFP^+^ cells in the midgut region of a single adult intestine. At least 2,000 cells were counted for each midgut. The sample size contained a minimum of 10 complete intestine samples for each genotype. The DAPI^+^ cells in each image were counted to estimate the total number of cells. The *P* values for between different genotypes were calculated using Student’s two-tailed *t* test.

### Lineage Tracing by esg^ts^ F/O Clone Induction

For clone induction in the CCR region, *UAS* lines were crossed to *w*; *esg-Gal4*, *tub-Gal80ts, UAS-GFP; UAS-FLP, Act* > *CD2* > *Gal4(UAS-GFP)/TM6B* flies. Crosses were set and cultured at 18°C until adulthood. After eclosure, 3-day-old females were collected and shifted to 29°C for 2 days, heat-shocked at 37°C for 45 min, recovered for 2 h at 29°C, and then heat-shocked at 37°C for 45 min again. Then, flies were maintained at 29°C for 5 days before dissection (procedure essentially adapted from Li et al., 2016).

### Immunohistochemistry

Primary antibodies used for immunostaining were mouse anti-Prospero 1:25 (Developmental Studies Hybridoma Bank, DHSB), mouse anti-β-gal 1:500 (DHSB), rabbit anti-cleaved *Drosophila* Dcp-1 1:100 (Cell Signaling), mouse anti-BrdU 1:100 (BD Biosciences), and mouse anti-Cut 1:20 (DHSB). Secondary antibodies used were goat Alexa Flour 594 conjugates at 1:200 dilutions (Invitrogen). Nuclei were stained using DAPI (Sigma). Confocal images were captured using Zeiss LSM 710, and Leica SP8 confocal imaging systems.

Adult flies were dissected in ice-cold PBS and fixed in 4% paraformaldehyde (Polysciences) for 1 h at room temperature. Fix removal was followed by 3 quick (1 min) washes and three long (45 min) washes using PBS with TritionX (1 × PBS + 0.5% BSA + 0.2% Triton X-100) and incubated with primary antibody at 4°C overnight. Samples were washed in PBST for 3 h, incubated with secondary antibody for 2 h at room temperature, and washed again in PBST as described above. Guts were subsequently stained with DAPI (2 μg/ml) and mounted in Vectashield antifade mounting media (Vector Labs). Each experiment was repeated at least three times and at least 10 guts were used for quantification of specific cell populations in each genotype sample.

### BrdU Labeling

BrdU staining was performed using standard methods with following modifications (Micchelli and Perrimon, 2006). Adult flies were cultured on standard fly media vials augmented with 6 μg/ml BrdU (Sigma) at 18°C. Flies were subsequently cultured at 29°C for 3 days for induction and transferred to BrdU + 5% glucose overnight to achieve maximal labeling. Adult flies were dissected and fixed in 4% paraformaldehyde followed by washes with PBST. Samples were treated with 2.5 M HCl for 10 min, neutralized with 100 mM sodium tetraborate buffer, and then incubated with primary antibody overnight at 4°C. Primary antibody was removed and the samples were washed in PBST. The samples were incubated for 2 h with secondary antibody, washed in PBST and DAPI and mounted with Vectashield antifade mounting media. Each experiment was repeated at least three times, and at least 10 guts were used for quantification of BrdU^+^ cells in each genotype sample.

### Image Processing and Analysis

Immunofluorescence images were processed using ImageJ software. Cells in the image were counted, keeping a rolling ball radius of 20–30 pixels and a Gaussian blur of 1 sigma (radius). Cells of size 5 μm^2^ to infinity were counted manually. Results of all experiments are plotted using GraphPad Prism 7.

### Transcript Profiling by Quantitative RT-PCR

Guts from flies of desired genotype and age (*n* = 20–40) were dissected and total RNA was extracted using the RNeasy kit as described by the manufacturer (Qiagen). One microgram of RNA was subsequently treated with RQ1 DNase (Promega). DNase-treated RNA was reverse transcribed using Improm-II reverse transcription system (Promega). Expression levels of candidate genes were quantified using ViiA 7 and QuantStudio 5 Real-Time PCR cycler system with SYBR green (Applied Biosystems). Each assay was performed in triplicate on at least three biological repeats. The expression levels of targets analyzed were calculated relative to GAPDH expression, using the ΔΔ*C*_*t*_ method using the formula: 2^∧^(-(ΔCt of experimental - ΔCt of control)). The results are presented as mean fold change with the standard error of the mean (SEM). *P* values were calculated using unpaired Student’s *t* test.

#### Primer Details

Oligonucleotides were designed with the help of DRSC FlyPrimerBank.

Oligonucleotide primers used for RT-PCR were as follows:

#### Ribosomal Protein 49 (*rp49*)

Forward: 5′-GCTAAGCTGTCGCACAAATG-3′.
Reverse: 5′-GTTCGATCCGTAACCGATGT-3′.

#### Glyceraldehyde 3-Phosphate Dehydrogenase (*gapdh*)

Forward: 5′-GTTCGGCCATAGCGAAAATCG-3′.
Reverse: 5′-GCGTCTCGTTGATAATCTCCG-3′.

#### Delta (*Dl*)

Forward: 5′-AATCCCATCCAGTTCCCCTTC-3′.
Reverse: 5′-ATTGCCGCTGTTGTTCGTATC-3′.

#### Supressor of Hairless (*Su(H)*)

Forward: 5′-AGCGCAACGACATGGTCAT-3′.
Reverse: 5′-GCGGTGGGCAAAAGAATCG-3′.

#### Escargot (*esg*)

Forward: 5′-ATGAGCCGCAGGATTTGTG-3′.
Reverse: 5′-CCTCCTCGATGTGTTCATCATCT-3′.

#### Defective Proventriculus (*dve*)

Forward: 5′-AAGAGGCGGCCCAATATCG-3′.
Reverse: 5′-GAGCGCCGTCTGAACAATCT-3′.

## Data Availability Statement

All datasets generated for this study are included in the article/Supplementary Material.

## Author Contributions

SM conceived the project, designed and performed the experiments, analyzed the data, and wrote the manuscript. PB and NO performed the experiments. SP analyzed the results and edited the manuscript. SG conceived the project and edited the manuscript. All authors contributed to the article and approved the submitted version.

## Conflict of Interest

The authors declare that the research was conducted in the absence of any commercial or financial relationships that could be construed as a potential conflict of interest.
