# Supplementary material for: Defective Proventriculus Regulates Cell Specification in the Gastric Region of Drosophila Intestine
Source: Front Physiol. 2020 Jul 14;11:711. doi: 10.3389/fphys.2020.00711 (PMC7372014; doi:10.3389/fphys.2020.00711)
Supplement: Supplementary file 1 [file Data_Sheet_1.PDF]

Supplementary Figures and Legends:

Figure S1:

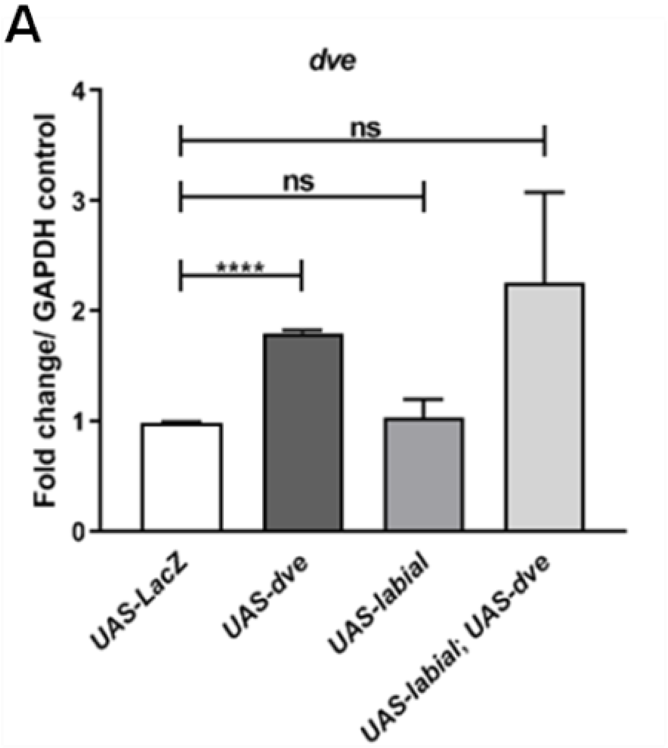

Supplementary Figure S1: Analysis of *dve* transcript levels in upon overexpression *labial* and/or *dve* in Esg^{+} cells in the midgut.

(A) Quantitative RT-PCR analyses of *dve* transcript levels in midguts from flies of the following genotypes: *esg^{ts}>UAS-lacZ* (Control), *esg^{ts}>UAS-dve,* *esg^{ts}>UAS-labial* and *esg^{ts}>UAS-labial,UAS-dve* after 3 days of induction at 29°C N=3, **p ≤0.01, and ns (not significant), p ≥0.05 calculated by student's two tailed t-test .

Figure S2:

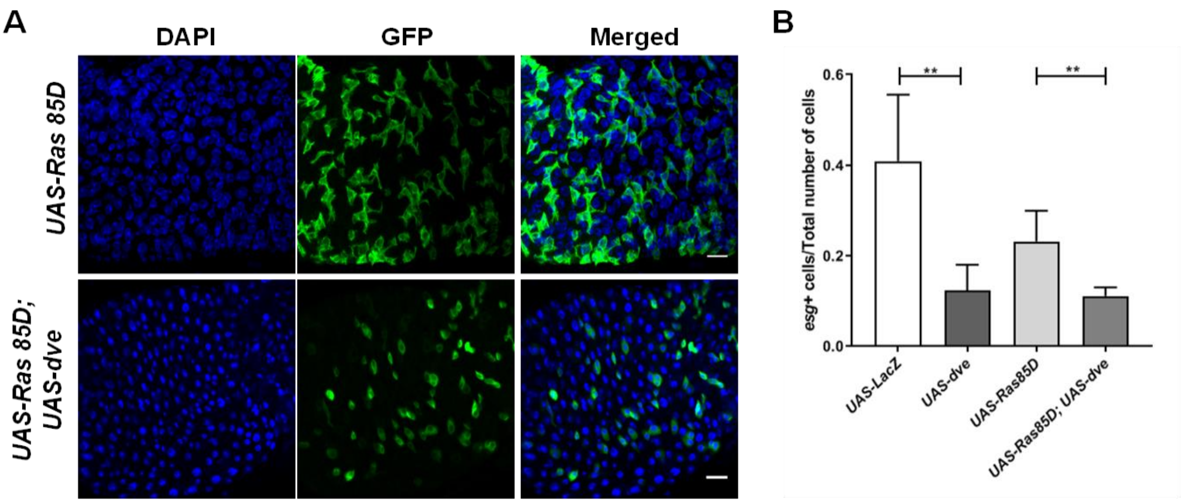

Esg>GFP, DNA

Supplementary Figure S2: *dve* overexpression in presence of activated EGF pathway also reduces the number of Esg^{+}(ISCs and EBs).

(A) Representative immunofluorescence images of midguts (DAPI-blue and GFP-green) from the flies of the following genotypes *esg^{ts}> UAS-ras 85D* and *esg^{ts}> UAS-ras 85D; UAS-dve*. Scale bar: 20µm. **(B)** Quantification of Esg^{+} cells (ISCs + EBs) in midguts of *esg^{ts}> UAS-ras 85D* and *esg^{ts}> UAS-ras 85D; UAS-dve* flies. Quantification of Esg^{+} cells in *esg^{ts}> UAS-lacZ* and *esg^{ts}> UAS-dve* as presented in Figure 1 has also been included in the graphs for ease of comparison of data. Data presented as mean $\pm$ SEM calculated from n =10 midgut, **p ≤0.01 (Student's two tailed t-test).

Figure S3:

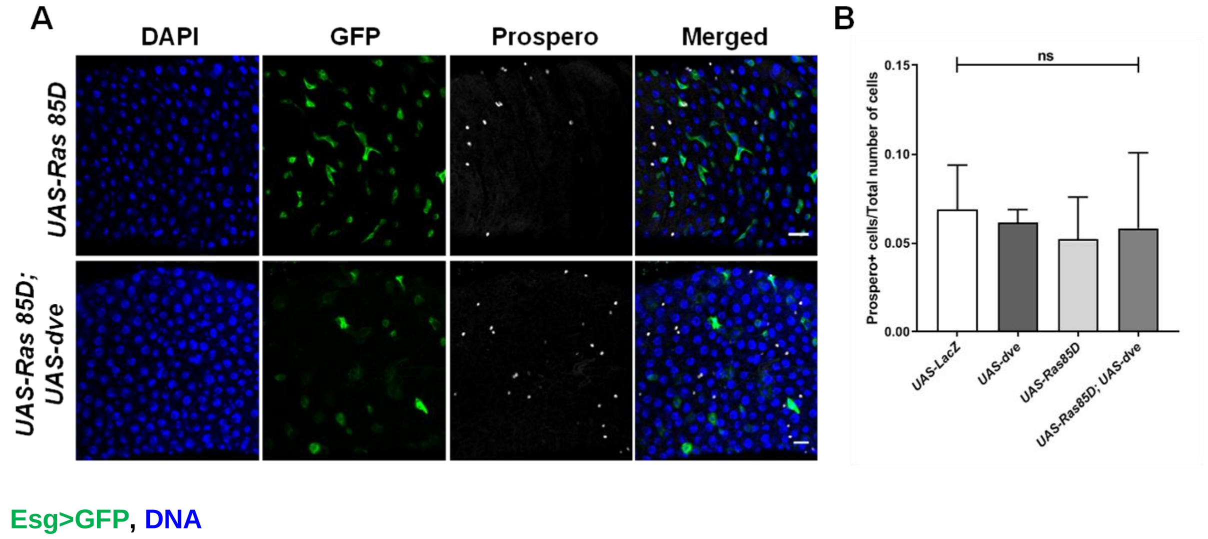

Supplementary Figure S3: Ectopic expression of *dve* does not change the number of differentiated enteroendocrine cells in presence of activated EGF pathway.

(A) Representative immunofluorescence images of midgut (DAPI-blue, GFP-green and Prospero-gray) *esg*^{ts}> *UAS-ras 85D* and *esg*^{ts}> *UAS-ras 85D; UAS-dve* flies. Scale bar: 20µm.

(B) Quantification of Prospero+ cells (ee) in midguts of *esg*^{ts}> *UAS-lacZ* (control), *esg*^{ts}> *UAS-dve, esg*^{ts}> *UAS-ras 85D* and *esg*^{ts}> *UAS-ras 85D; UAS-dve/+* flies. Quantification of Propero^{+} cells in *esg*^{ts}> *UAS-lacZ* and *esg*^{ts}> *UAS-dve* as presented in Figure 4 has also been included in the graphs for ease of comparison of data. Data presented as mean $\pm$ SEM, from n=10 midgut, ns: not significant, p $\geq$0.05 (Student's two tailed t-test).
